# Mass spectrometry-based analysis of formalin-fixed, paraffin-embedded distal cholangiocarcinoma identifies stromal thrombospondin-2 as a potential prognostic marker

**DOI:** 10.1186/s12967-020-02498-3

**Published:** 2020-09-04

**Authors:** Johannes Byrling, Theresa Kristl, Dingyuan Hu, Indira Pla, Aniel Sanchez, Agata Sasor, Roland Andersson, György Marko-Varga, Bodil Andersson

**Affiliations:** 1 Department of Clinical Sciences Lund, Surgery, Lund University, and Skåne University Hospital, Lund, Sweden; 2 Department of Biomedical Engineering, Clinical Protein Science and Imaging, Lund University, Lund, Sweden; 3 Department of Clinical Sciences Lund, Pathology, Lund University, and Skåne University Hospital, Lund, Sweden

**Keywords:** Distal cholangiocarcinoma, Biliary tract cancer, Mass spectrometry, Parallel reaction monitoring, Biomarker, Stroma, Thrombospondin-2

## Abstract

**Background:**

Distal cholangiocarcinoma is an aggressive malignancy with a dismal prognosis. Diagnostic and prognostic biomarkers for distal cholangiocarcinoma are lacking. The aim of the present study was to identify differentially expressed proteins between distal cholangiocarcinoma and normal bile duct samples.

**Methods:**

A workflow utilizing discovery mass spectrometry and verification by parallel reaction monitoring was used to analyze surgically resected formalin-fixed, paraffin-embedded samples from distal cholangiocarcinoma patients and normal bile duct samples. Bioinformatic analysis was used for functional annotation and pathway analysis. Immunohistochemistry was performed to validate the expression of thrombospondin-2 and investigate its association with survival.

**Results:**

In the discovery study, a total of 3057 proteins were identified. Eighty-seven proteins were found to be differentially expressed (q < 0.05 and fold change ≥ 2 or ≤ 0.5); 31 proteins were upregulated and 56 were downregulated in the distal cholangiocarcinoma samples compared to controls. Bioinformatic analysis revealed an abundance of differentially expressed proteins associated with the tumor reactive stroma. Parallel reaction monitoring verified 28 proteins as upregulated and 18 as downregulated in distal cholangiocarcinoma samples compared to controls. Immunohistochemical validation revealed thrombospondin-2 to be upregulated in distal cholangiocarcinoma epithelial and stromal compartments. In paired lymph node metastases samples, thrombospondin-2 expression was significantly lower; however, stromal thrombospondin-2 expression was still frequent (72%). Stromal thrombospondin-2 was an independent predictor of poor disease-free survival (HR 3.95, 95% CI 1.09–14.3; P = 0.037).

**Conclusion:**

Several proteins without prior association with distal cholangiocarcinoma biology were identified and verified as differentially expressed between distal cholangiocarcinoma and normal bile duct samples. These proteins can be further evaluated to elucidate their biomarker potential and role in distal cholangiocarcinoma carcinogenesis. Stromal thrombospondin-2 is a potential prognostic marker in distal cholangiocarcinoma.

## Introduction

Cholangiocarcinoma (CCA), an epithelial tumor arising along the biliary tree, is a low- incidence malignancy accounting for approximately 3% of all gastrointestinal cancers [1]. CCA is a notably aggressive malignancy with a current overall 5-year survival rate of less than 5% [2]. Surgical resection is the only treatment that offers curative potential. However, an invasive growth pattern and the absence of early symptoms contribute to presentation with metastatic disease in a majority of patients [3]. Based on its anatomical location along the biliary tree, CCA is classified as intrahepatic (iCCA) or extrahepatic (eCCA) which is further divided into the perihilar (pCCA) and distal (dCCA) subtypes [4]. Anatomically, dCCA is located between the insertion of the cystic duct and the ampulla of Vater [4, 5].

Mounting evidence points to a significant difference between the different subtypes with regards to not only clinical management but also tumor biology and molecular characteristics [3, 6, 7]. However, the majority of previous biomarker studies do not take this subclassification into account [3, 8]. Currently, only one biomarker, namely, plasma carbohydrate antigen 19-9 (CA 19-9) is in clinical use for the diagnosis of CCA. The sensitivity and specificity of CA 19-9 are not sufficient to allow screening of unselected patient populations [9].

A large effort has elucidated the spectra of genomic and transcriptomic alterations associated with malignancy along the biliary tree [6, 7]. However, changes at the mRNA level do not necessarily correlate with changes at the protein level [10, 11], and studies of the proteomic alterations that develop during carcinogenesis can provide complementary information and help identify new biomarkers [12, 13]. Modern mass spectrometry (MS) platforms utilizing online liquid-phase separation with tandem MS (LC–MS/MS) have been used for protein identification and quantification in human sample cohorts, enabling quantitative comparison of thousands of proteins [13–15]. Traditionally, MS-based proteomics has been performed using fresh frozen tissues. However, it has been demonstrated that the more widely available formalin-fixed, paraffin-embedded (FFPE) tissues can also be utilized successfully [16, 17].

A discovery LC–MS/MS experiment can identify a large number of differentially expressed proteins (DEPs) between the investigated biological conditions providing information regarding proteomic alterations associated with malignancy as well as biomarker candidates. However, few identified potential biomarkers in cancer research have been able to progress to clinical utilization, in part due to the difficulty in validating the large number of biomarkers [18]. Parallel reaction monitoring (PRM) is an MS technique developed for targeted quantification [19, 20]. PRM has been shown to provide improved quantitative precision and to be able to quantify proteins at lower concentrations than a typical discovery LC–MS/MS experiment. Thus, PRM can be used for large scale verification of proteins identified from discovery experiments [21, 22].

The aim of the present study was to use a combined discovery LC–MS/MS and PRM-verification workflow to analyze resected dCCA and normal bile duct FFPE samples to identify DEPs. As a secondary aim, further validation of thrombospondin-2 (THBS2) expression was performed using immunohistochemistry (IHC).

## Materials and methods

### Study population

All consecutive patients who underwent surgery with curative intent for dCCA at the Department of Surgery, Skåne University Hospital, Lund and Malmö, between January 2000 and December 2015 were identified from hospital records. Only patients with no neoadjuvant treatment and no 30-day mortality were included. Sixty-four patients were identified. FFPE materials from 59 patients were available and subsequently retrieved from the local biobank at the Department of Pathology, Skåne University Hospital, Lund and Malmö. All samples underwent histopathological reevaluation by an experienced gastrointestinal pathologist (A.S) blinded to the original assessment. The cohort has been previously described [23, 24]. Staging was based on the American Joint Committee on Cancer (AJCC) 7th edition [5]. R1 resection was defined as cancer growth < 1 mm from the resection margin. Demographical and clinical data were retrospectively acquired through patients’ medical records. Patients who received at least 3 cycles of postoperative chemotherapy were coded as having received adjuvant treatment. The clinicopathological data of the cohort are presented in Table 1. Patients were postoperatively followed for up to 5 years. Survival analysis was censored at 5 years after surgery for event-free patients. Survival status was recorded in September 2019. Disease-free survival (DFS) was defined as the time until clinical diagnosis of dCCA recurrence or death from any cause. Overall survival (OS) was defined as the time until death of any cause.

**Table 1:** Clinicopathological data in the distal cholangiocarcinoma cohort (N = 59)

Variable	N	n (%), mean ± SD
Age	59	67 ± 8.1
Sex	59	
Female		20 (34%)
Adjuvant therapy		
Yes	58	30 (52%)
Tumor size (mm)	59	29 ± 9.6
Tumor differentiation	59	
High		0
Intermediate		16 (27%)
Low		43 (73%)
T stage	59	
I		2 (3.4%)
II		0
III		56 (95%)
IV		1 (1.7%)
Lymph node metastases	58	
Present		40 (69%)
AJCC stage	58	
IA		1 (1.7%)
IB		0
IIA		17 (29%)
IIB		39 (67%)
III		1 (1.7%)
R1 resection	59	
Present		34 (58%)
Lymph vessel invasion	57	
Present		42 (74%)
Nerve invasion	58	
Present		48 (83%)
Blood vessel invasion	58	
Present		37 (64%)
Adipose invasion	59	
Present		48 (81%)

*AJCC* American Joint Committee on cancer, *R1* non-radical resection, *SD* standard deviation, *T stage* Tumor stage

The control tissues were identified from a prospective database of pancreaticobiliary surgery maintained at the Department of Surgery, Skåne University Hospital, Lund and Malmö. Patients who underwent pancreaticoduodenectomy due to a benign diagnosis without proximity to the distal bile duct were identified.

### MS tissue area selection

Samples were sectioned into 10-μm sections. Hematoxylin and eosin (H&E)-stained slides were used to mark the areas of interest in each slide for macrodissection. Areas of interest in the dCCA samples were defined as areas containing only tumor epithelium and intratumoral stromal compartment without any adjacent tissues. The normal bile duct tissues were retrieved from the region between the cystic duct and the ampulla of Vater. The bile duct tissues were histopathologically evaluated, and any sample with dysplasia or inflammation of the bile duct was excluded. The macrodissected area was located from the epithelial lining and up to, but not including the adventitia or pancreatic parenchyma. Macrodissection was carried out using a scalpel with the marked H&E slides as a template for serial sections. An area corresponding to approximately 3 full slides was utilized when available. In some samples with scarce material the maximum available amount was retrieved although it did not correspond to 3 full sections. The material was stored at 4 °C.

### MS sample preparation

Twenty dCCA samples from the ten patients each with the worst and best OS were selected for the discovery study. The selection was performed to allow for additional comparison between prognostic groups that did not reveal significant results (data not shown).

All samples were processed in a manner blinded to patient identity and outcome. Preparation of all samples was performed in parallel. For deparaffinization and protein retrieval, macrodissected samples were incubated in 1 ml of EnVision FLEX Target Retrieval Solution (High pH) (K8004, Dako, Glostrup, Denmark) at a dilution of 1:50 at 97 °C for 10 min. Samples were then centrifuged at 14,000 rcf at 4 °C for 10 min. The supernatants were removed and the deparaffinization steps were repeated. Samples were resuspended in 150 μl of 500 mM Tris–HCL (pH 8), followed by transfer to a new tube and incubation at 90 °C for 1 h. Then, 150 μl of 6 M guanidine-HCl in 50 mM ammonium bicarbonate (AMBIC) was added, followed by probe sonication for 2 × 4 min on ice. The samples were then centrifuged at 14,000 rcf for 10 min at 24 °C, and the supernatants were transferred to a new tube. Proteins were reduced by the addition of 6 μl of 1 M dithiothreitol and incubated for 1 h at 56 °C. Alkylation was performed by the addition of 20 μl of 1 M iodoacetamide for 30 min in dark. Precipitation was carried out overnight using pure ethanol at a ratio (v/v) (sample:ethanol) of 1:9, and the samples were stored at − 20 °C. Following centrifugation at 14,000 rcf for 15 min at 4 °C the supernatants were carefully discarded, and the pellets were resuspended in 100 μl of 50 mM AMBIC. Protein determination was carried out using the Micro BSA Protein Assay Kit (Thermo Fisher Scientific, Rockford, IL, USA) in accordance with the manufacturer’s instructions. Digestion was carried out using sequencing-grade trypsin (Promega, Madison, WI, USA) at a ratio (w/w) (trypsin:protein) of 1:50, followed by incubation at 37 °C overnight. The samples were dried using centrifugal evaporation and resuspended in 0.1% formic acid. Peptide determination was performed according to the manufacturer’s instructions using the Pierce Quantitative Colorimetric Peptide Assay (Thermo Fisher Scientific, Rockford, IL, USA). The samples were then spiked with Pierce Peptide Retention Time Calibration Mixture (Thermo Fisher Scientific, Rockford, IL, USA) to evaluate chromatographic performance. A total of 25 fmol of Pierce Retention Time Calibration Mixture was added to every μg of peptide in the sample and the samples were diluted to a final concentration of 0.25 μg/μl in 0.1% formic acid for injection.

### Liquid chromatography conditions

The liquid chromatography (LC) instrument used was an EASY-nLC (Thermo Fisher Scientific, Rockford, IL, USA). Previously prepared material from one patient was measured repeatedly at regular intervals as a quality control. One microgram of sample was injected and measured with a flow rate of 300 nL/minute, and a two-column setup consisting of an Acclaim PepMap RSLC column (75 µm × 25 cm) as the analytical column and Acclaim PepMap 100 column (75 µm × 2 cm) as the precolumn (both from Thermo Fisher Scientific, Rockford, IL, USA) was used for the separation. The LC gradient was created using solvent A (0.1% formic acid) and solvent B (0.1% formic acid in acetonitrile). Based on manual evaluation of the number of peptides eluted at different time points, a nonlinear gradient was developed. The nonlinear gradient started at 5% B and increased to 22% B at 95 min and 36% B at 150 min. All measurements in the discovery study were performed in technical duplicates. Samples were measured in a randomized order.

### MS conditions

The samples were analyzed using a Q Exactive Plus mass spectrometer (Thermo Fisher Scientific, Rockford, IL, USA). The equipped ion source was an EASY-Spray (Thermo Fisher Scientific, Rockford, IL, USA). The system was operated in positive mode, data-dependent acquisition (DDA) was used. For peptide identification, a full MS survey scan was collected in the Orbitrap. Fifteen data-dependent higher energy collision dissociation MS/MS scans of the most intense precursors were performed.

The spray voltage was set to 1.75 kV, and the capillary temperature was 300 °C. Moreover, the S-lens radiofrequency level was fixed at 50. MS1 survey scans of the eluting peptides were executed with a resolution of 70,000, recording a window between m/z 350.0 and 1800. The automatic gain control (AGC) target was set to 1 × 10^6^ and the maximum injection time was 100 ms. The normalized collision energy (NCE) was set to 25.0% for all scans. The resolution of the data-dependent MS^2^ scans was fixed to 35,000, and the values for the AGC target were set to 1 × 10^6^ with a maximum injection time of 120 ms.

### Protein identification and quantification

The software Proteome Discoverer (PD) (version 1.4) (Thermo Fisher Scientific, Rockford, IL, USA) was used for protein identification. The selection of spectra employed the following settings: minimal and maximal precursor mass, 350 and 5000 Da, respectively; and signal-to-noise threshold of 1.5. Parameters for Sequest HT searches were set as follows: precursor mass tolerance, 10 ppm; fragment mass tolerance, 0.02 Da; trypsin; 1 missed cleavage site; UniProt human database; dynamic modification; oxidation (+ 15.995 Da; (M, P)); static modification; carbamidomethyl (+ 57.021 Da; (C)). A percolator was used for the processing node and the false discovery rate (FDR) was set to 1%. Proteins were identified using at least two peptides. A precursor ion area detector was used for quantification, and each protein was quantified from the average area of the 3 most abundant peptides identified for that particular protein. To increase the number of candidate proteins for PRM validation, we also performed protein identification and quantification using two alternative software programs, MaxQuant and OpenMS. The settings used for each software program are presented in Additional file 1.

### PRM verification

To verify the DEPs identified from the discovery study, a targeted proteomic study was performed using PRM. Selection of suitable proteins for inclusion in the PRM study was performed through evaluation of the DEPs after quantification using PD, MaxQuant and OpenMS. In addition, proteins successfully quantified in only dCCA samples or controls respectively were considered. All proteins were submitted to a literature review, and proteins previously found to be dysregulated in CCA or with a known association to cancer biology were prioritized for inclusion. Peptide selection was based on the data from the PD evaluation of the discovery study. Only peptides with no missing cleavages were included. Peptide properties (charge state, precursor m/z, retention time) were extrapolated from PD.

Materials from the 20 dCCA samples used in the discovery study and 13 controls were prepared anew (additional material was available from 8 control samples analyzed in the discovery study, and 5 new samples were added). Sample preparation was performed in accordance with the same protocol utilized in the discovery study. The same LC instrumentation and settings and MS instrumentation used in the discovery study were used for the PRM study. Measurements were performed in technical triplicates when possible. For samples with a low amount of material, one or two technical replicates were deemed acceptable. For the measurements, 1 µg of sample was injected into the instrument. PRM acquisition was performed without retention time scheduling over the complete chromatographic run. The MS^2^ resolution was set at 70,000, with the AGC target set to 5 × 10^5^ and a maximum injection time of 70 ms. The chromatic peak width was 30 s. The NCE was set to 26.0% and the isolation window was 2.0 m/z. MS1 ion chromatogram extraction and relative quantification were performed using Skyline [25].

### IHC

The entire dCCA cohort (N = 59), including paired lymph node metastases in available samples (N = 26) and control samples (N = 10), was selected for IHC validation. FFPE samples were sectioned to 4 µm, deparaffinized in xylene and rehydrated in graded ethanol solutions. Antigen retrieval was performed using EnVision FLEX Target Retrieval Solution (low pH) (K8005, Dako, Glostrup, Denmark) at 97 °C for 20 min using an automated PT Link (Dako, Glostrup, Denmark). Endogenous peroxidase activity was quenched with 0.3% hydrogen peroxide and 1% methanol in phosphate-buffered saline for 10 min at room temperature. After blocking with 5% goat serum and avidin/biotin blocking kit (SP-2001, Vector Labs, Burlingame, CA, USA) the sections were incubated with primary polyclonal rabbit anti-THBS2 antibody diluted 1:100 (Ab112543, Abcam, Cambridge, UK) overnight at 4 °C. A biotinylated goat anti-rabbit secondary antibody diluted 1:200 (BA-1000, Vector Labs, Burlingame, CA, USA) was added to the slides and incubated for 1 h at room temperature. The sections were subsequently incubated with avidin–biotin-peroxidase complex Vectastain Elite ABC (PK-6100, Vector Laboratories, Burlingame, CA, USA) for 30 min. For color development the slides were exposed to the chromogen 3,3´diaminobenzidine (SK-4100, Vector Laboratories, Burlingame, CA, USA) for 8 min. Following counterstaining with Mayer’s hematoxylin, the sections were dehydrated with graded ethanol solutions and mounted with Pertex. Omission of primary antibody was used as a negative control. Placental tissue sections were included as positive controls.

IHC staining was evaluated by an experienced gastrointestinal pathologist (A.S). The predominant staining pattern in the intratumoral area was evaluated.

Staining was reviewed under light microscopy at 200× magnification. Given that both epithelial and stromal immunoreactivity was evident, separate scoring was performed for both compartments. Expression in > 10% of cells was recorded as positive expression. The staining intensity was scored as 0 (negative), 1+ (low), 2+ (moderate) or 3+ (strong).

### Statistical analysis and bioinformatics

Statistical processing of the MS data from the discovery and PRM study was done using Perseus [26] and GraphPad Prism 8. In the discovery study, the mean value of technical replicates was used. The data was filtered, only proteins quantified in at least 50% of the dCCA and control samples were used for further analysis. Values were log2 transformed and normalized by subtracting the median protein intensities in each sample. Missing values were replaced by imputing random numbers drawn from a normal distribution similar to the measured data (width = 0.3, downshift = 0). DEPs between the dCCA samples and controls were identified using a two-tailed t-test followed by multiple testing permutation-based FDR. Finally, proteins with a q value (adjusted p-value) < 0.05 (FDR 5%) and fold change (FC) ≥ 2 or ≤ 0.5 were considered significantly differentially expressed.

For statistical evaluation of the PRM data, the mean values of technical replicates were used. All data were log2 transformed and normalization to the average value of peptides quantifying the housekeeping proteins glyceraldehyde 3-phosphate dehydrogenase (GAPDH) (GALQNIIPASTGAAK, LISWYDNEFGYSNR) and tubulin beta chain (TUBB) (ISVYYNEATGGK) in each sample was performed. Group comparison of DEPs was performed identically to the discovery study. Gene ontology (GO) [27] classification was performed using the PANTHER online bioinformatics tool [28]. The PANTHER overrepresentation test of functional annotation and pathways was used. In addition the DAVID bioinformatics tool [29] was used to perform additional pathway analysis against the KEGG [30] and REACTOME [31] databases. All enrichment analysis were performed against the background of the total number of proteins identified in the study. FDR adjustment for multiple testing was employed, and a q < 0.05 (FDR 5%) was used to indicate significance in all enrichment tests. Protein–protein interactions were investigated using the STRING online bioinformatics tool [32]. An interaction score ≥ 0.7 (high confidence) was required for inclusion in the model.

Stata MP statistical package version 14.2 was used for analysis of IHC data. Comparison of continuous baseline parameters was performed with the t-test or Mann–Whitney U test as appropriate. Categorical data were compared using Fisher’s exact test. The exact McNemar’s test was used to compare expression in primary tumors and paired lymph node metastases. Spearman correlation was used to compare expression in dCCA epithelial and stromal compartments. The survival rates were estimated using the Kaplan–Meier method and log-rank test. Multivariable Cox proportional hazards regression was performed to adjust for confounders. The multivariable model included age, sex, tumor differentiation, R1 resection, lymph node metastases and adjuvant treatment as covariables. The assumption of proportional hazards was evaluated using Schoenfeld residuals. All tests were two-tailed and a p-value of 0.05 was used to indicate significance.

## Results

### Discovery study

**Fig. 1:**
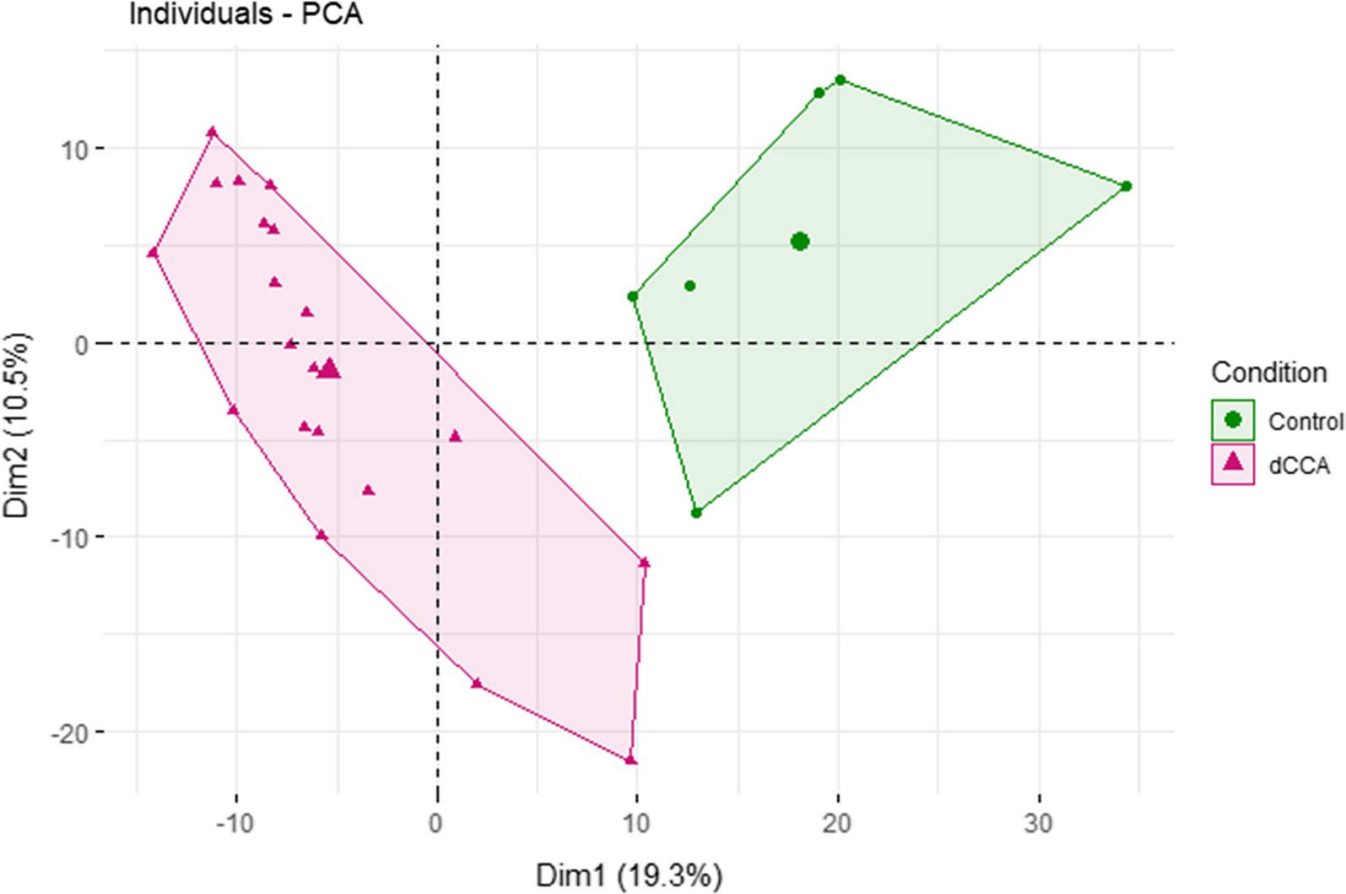
Principal component analysis of the 836 proteins that were quantified in atleast 50% of distal cholangiocarcinoma samples (pink) and controls (green)

A workflow including discovery LC–MS/MS followed by PRM verification using macrodissected archived FFPE samples was used to identify DEPs between dCCA and controls. For the discovery study materials from 20 dCCA samples and 10 controls were prepared for analysis. Four control samples could not be measured due to a low protein yield. The clinicopathological data of the patients whose samples were analyzed are presented in Additional file 2. In total, 3037 proteins were identified (Additional file 3). In the dCCA samples, 2967 proteins were identified, and in the controls, 1501 proteins were identified. After removal of all proteins with quantifiable values in fewer than 50% of the samples in each group, a total of 836 proteins remained. Principal component analysis (PCA) of the 836 proteins reveled the evident separation of the groups. Components 1 and 2 accounted for 19.1% and 9.9% of total variation, respectively (Fig. 1). A total of 87 DEPs (q < 0.05, FC ≥ 2 or ≤ 0.5) were identified (Additional file 4). Of the 87 DEPs, 31 proteins were found to be upregulated in the dCCA samples relative to controls and 56 were downregulated. In addition to quantification using PD, quantification with MaxQuant and OpenMS generated 109 and 135 DEPs, respectively (Additional file 4).

### Bioinformatic analysis

**Fig. 2:**
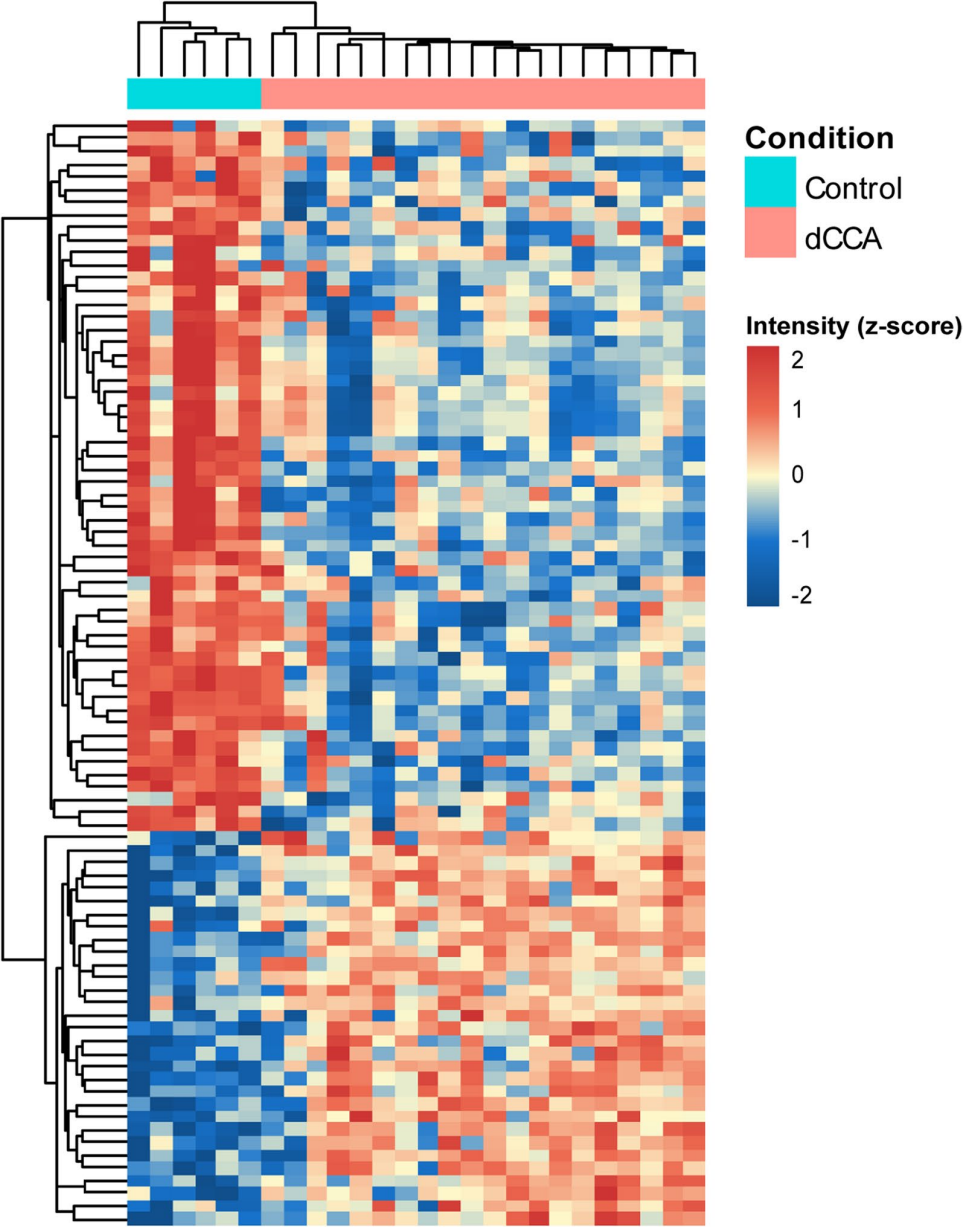
Heatmap showing the results of two-way unsupervised hierarchical clustering of the 87 proteins found to be differentially expressed (q < 0.05, FC ≥ 2 or ≤ 0.5) between the distal cholangiocarcinoma samples (pink) and controls (teal)

**Fig. 3:**
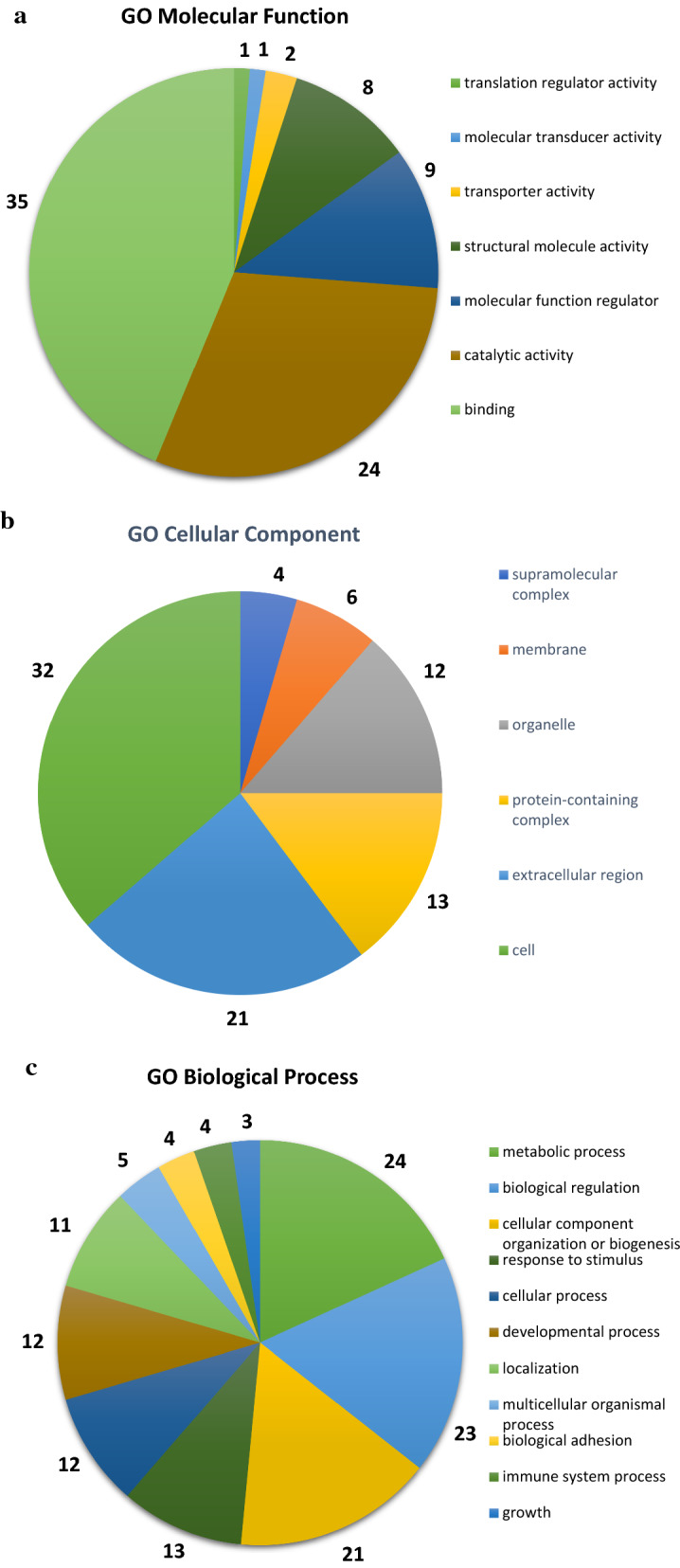
Gene ontology classification of the 87 differentially expressed proteins (q < 0.05, FC ≥ 2 or ≤ 0.5) between the distal cholangiocarcinoma samples and controls using the PANTHER database, with the number of genes presented. **a** Molecular function. **b** Cellular component. **c** Biological process

**Fig. 4:**
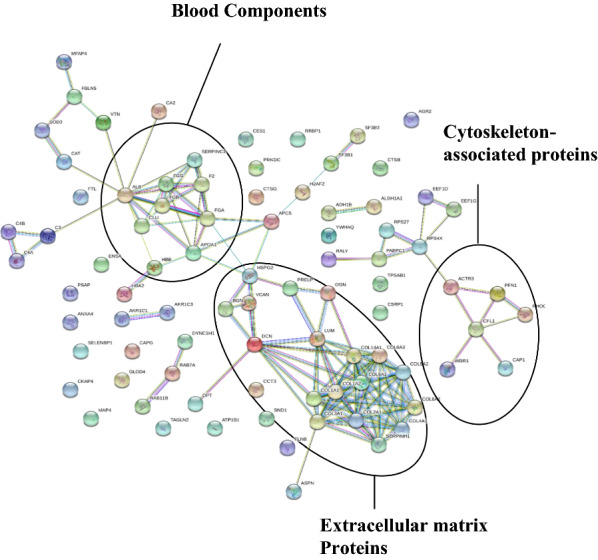
Protein–protein interactions of the 87 differentially expressed proteins (q < 0.05, FC ≥ 2 or ≤ 0.5) between the distal cholangiocarcinoma samples and controls obtained using the STRING database. Discernable clusters of extracellular matrix proteins, blood components and cytoskeleton-associated proteins are highlighted

Two-way unsupervised hierarchical clustering was applied to the 87 DEPs, and the results were visualized in a heat-map (Fig. 2). Two clusters of proteins that clearly separated dCCA and control samples were evident. GO analysis of the DEPs revealed the molecular functions in which the DEPs where most frequently involved to be binding, catalytic activity and molecular function regulation (Fig. 3a). The cellular components in which the DEPs were most frequently involved were cell, extracellular region and protein-containing complex (Fig. 3b). The biological processes in which the DEPs were most frequently involved were metabolic process, biological regulation and biological adhesion (Fig. 3c). PANTHER-overrepresentation test was performed on the 87 DEPs. No statistically significant enrichment with regards to molecular function or biological process was seen. When cellular components were analyzed, collagen trimer, extracellular space, extracellular matrix (ECM) and its lineage parent extracellular region and extracellular region part were significantly enriched in the DEPs. PANTHER pathway analysis revealed that the integrin signaling pathway was significantly enriched in the DEPs. KEGG pathway analysis revealed that protein digestion and absorption and ECM-receptor interaction were significantly enriched in the DEPs. When pathway enrichment was analyzed with REACTOME, ECM proteoglycans, integrin cell surface interactions, collagen degradation, assembly of collagen fibrils and other multimeric structures, collagen biosynthesis and modifying enzymes, neural cell adhesion molecule 1 interactions, ECM organization, signaling by platelet-derived growth factor and scavenging by class A receptors were found to be significantly enriched in the DEPs. Detailed results of the enrichment analysis are presented in Additional file 5. The STRING-database was employed to identify protein–protein interactions within the DEPs. In tota1, 46 protein interactions were found, and these interactions were significantly enriched based on the given nodes (P < 0.001). A complex pattern of protein–protein interactions was found, and clusters of ECM proteins, blood components and cytoskeleton-associated proteins could be discerned (Fig. 4).

### Targeted verification

For PRM verification, in total 33 patient samples were selected (20 dCCA samples and 13 controls). Minute number of samples, many with low protein yields allowed for 25 samples to comply with the PRM analysis criteria, comprising 16 dCCA and 9 control samples.

Clinicopathological data of the samples analyzed by PRM are presented in Additional file 2. The median coefficient of variation (CV)% of technical replicates for each protein ranged from 3 to 23%. A total of 170 peptides mapping to 91 proteins, including the housekeeping proteins GAPDH and TUBB, were included in the PRM spectral library (Additional file 6). A total of 122 peptides mapping to 79 proteins were successfully quantified (Additional file 7).

**Fig. 5:**
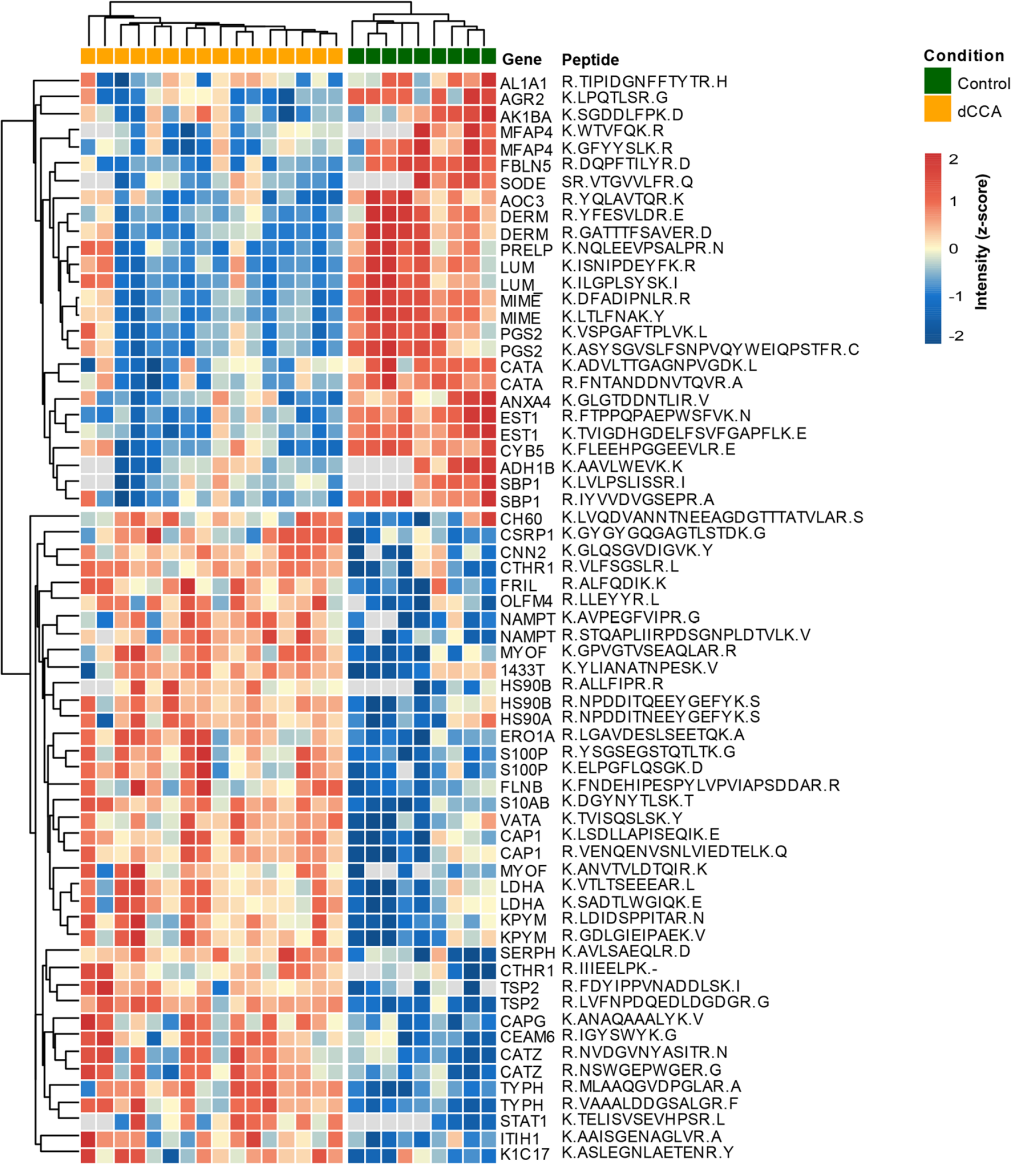
Heatmap showing the results of two-way unsupervised hierarchical clustering of the 46 proteins found to be differentially expressed by PRM verification (q < 0.05, FC ≥ 2 or ≤ 0.5) between the distal cholangiocarcinoma samples (yellow) and controls (green) respectively

In total, 65 different peptides were found to be differentially expressed (q < 0.05, FC ≥ 2 or ≤ 0.5). These peptides mapped to 46 proteins, of which 19 were identified as differentially expressed by 2 different peptides, and 27 proteins were identified as differentially expressed based on 1 peptide. Thirty-nine peptides mapping to 28 proteins were found to be upregulated, and 26 peptides mapping to 18 proteins were found to be downregulated when dCCA samples were compared to controls (Table 2). Previous associations between the identified proteins and CCA are presented in Table 2. Hierarchical clustering revealed a good separation between dCCA and controls based on the DEPs from the PRM verification (Fig. 5).

**Table 2:** Proteins found to be differentially expressed in the PRM analysis (q < 0.05, FC ≥ 2 or ≤ 0.5) between the distal cholangiocarcinoma samples and controls

#	Accession	Gene	Protein Name	Peptide Sequence	Values Cancer	Values Control	q-value	FC Ca/Co	Literature CCA
A									
1	P35442	TSP2	Thrombospondin-2	LVFNPDQEDLDGDGR	16	9	< 0.0001	29.3	
2	P35442	TSP2	Thrombospondin-2	FDYIPPVNADDLSK	16	6	< 0.0001	8.3	
3	Q6UX06	OLFM4	Olfactomedin-4	LLEYYR	16	8	< 0.0001	7.8	[71, 72]
4	P25815	S100P	Protein S100-P	YSGSEGSTQTLTK	16	9	< 0.0001	7.3	[63, 64]
5	P40199	CEAM6	Carcinoembryonic antigen-related cell adhesion molecule 6	IGYSWYK	16	9	0.0007	7.2	[65]
6	P25815	S100P	Protein S100-P	ELPGFLQSGK	16	8	< 0.0001	6.4	[63, 64]
7	Q04695	K1C17	Keratin, type I cytoskeletal 17	ASLEGNLAETENR	16	9	0.0044	5.5	[66, 67]
8	Q99439	CNN2	Calponin-2	GLQSGVDIGVK	16	8	< 0.0001	4.8	
9	Q96CG8	CTHR1	Collagen triple helix repeat-containing protein 1	VLFSGSLR	16	9	< 0.0001	4.5	
10	P08238	HS90B	Heat shock protein HSP 90-beta	NPDDITQEEYGEFYK	16	9	< 0.0001	4.2	[70]
11	P19971	TYPH	Thymidine phosphorylase	MLAAQGVDPGLAR	16	9	< 0.0001	4.1	[68, 69]
12	P31949	S10AB	Protein S100-A11	DGYNYTLSK	16	9	< 0.0001	3.8	[96]
13	P19971	TYPH	Thymidine phosphorylase	VAAALDDGSALGR	16	9	< 0.0001	3.6	[68, 69]
14	P19827	ITIH1	Inter-alpha-trypsin inhibitor heavy chain H1	AAISGENAGLVR	16	9	0.0030	3.5	
15	Q96HE7	ERO1A	ERO1-like protein alpha	LGAVDESLSEETQK	16	9	< 0.0001	3.0	
16	Q01518	CAP1	Adenylyl cyclase-associated protein 1	VENQENVSNLVIEDTELK	16	9	< 0.0001	2.9	
17	Q9UBR2	CATZ	Cathepsin Z	NVDGVNYASITR	16	9	0.0004	2.7	
18	P50454	SERPH	Serpin H1	AVLSAEQLR	16	9	< 0.0001	2.7	[97]
19	P21291	CSRP1	Cysteine and glycine-rich protein 1	GYGYGQGAGTLSTDK	16	9	0.0018	2.7	
20	Q9UBR2	CATZ	Cathepsin Z	NSWGEPWGER	16	9	0.0007	2.6	
21	P02792	FRIL	Ferritin light chain	ALFQDIK	16	9	0.0002	2.6	
22	Q01518	CAP1	Adenylyl cyclase-associated protein 1	LSDLLAPISEQIK	16	9	< 0.0001	2.5	
23	P42224	STAT1	Signal transducer and activator of transcription 1-alpha/beta	TELISVSEVHPSR	14	4	0.0033	2.5	[98]
24	Q9NZM1	MYOF	Myoferlin	ANVTVLDTQIR	16	6	0.0020	2.4	
25	Q96CG8	CTHR1	Collagen triple helix repeat-containing protein 1	IIIEELPK	16	6	0.0020	2.4	
26	P00338	LDHA	L-lactate dehydrogenase A chain	SADTLWGIQK	16	9	< 0.0001	2.4	[99, 100]
27	P00338	LDHA	L-lactate dehydrogenase A chain	VTLTSEEEAR	16	9	< 0.0001	2.3	[99, 100]
28	O75369	FLNB	Filamin-B	FNDEHIPESPYLVPVIAPSDDAR	16	9	0.0005	2.3	
29	P43490	NAMPT	Nicotinamide phosphoribosyltransferase	STQAPLIIRPDSGNPLDTVLK	15	8	< 0.0001	2.2	
30	Q9NZM1	MYOF	Myoferlin	GPVGTVSEAQLAR	16	9	< 0.0001	2.2	
31	P40121	CAPG	Macrophage-capping protein	ANAQAAALYK	16	9	0.0006	2.2	[87]
32	P38606	VATA	V-type proton ATPase catalytic subunit A	TVISQSLSK	16	9	< 0.0001	2.2	
33	P14618	KPYM	Pyruvate kinase PKM	LDIDSPPITAR	16	9	< 0.0001	2.1	[101]
34	P08238	HS90B	Heat shock protein HSP 90-beta	ALLFIPR	14	5	0.0021	2.1	[70]
35	P27348	1433 T	14–3-3 protein theta	YLIANATNPESK	16	9	0.0078	2.1	[102, 103]
36	P07900	HS90A	Heat shock protein HSP 90-alpha	NPDDITNEEYGEFYK	16	9	0.0092	2.1	[70]
37	P43490	NAMPT	Nicotinamide phosphoribosyltransferase	AVPEGFVIPR	16	7	< 0.0001	2.1	
38	P14618	KPYM	Pyruvate kinase PKM	GDLGIEIPAEK	16	9	< 0.0001	2.1	[101]
39	P10809	CH60	60 kDa heat shock protein, mitochondrial	LVQDVANNTNEEAGDGTTTATVLAR	16	9	0.0408	2.0	[104]
B									
1	P00352	AL1A1	Retinal dehydrogenase 1	TIPIDGNFFTYTR	16	9	0.0457	0.46	[105]
2	P04040	CATA	Catalase	ADVLTTGAGNPVGDK	16	9	< 0.0001	0.34	[106]
3	P04040	CATA	Catalase	FNTANDDNVTQVR	16	9	< 0.0001	0.32	[106]
4	P09525	ANXA4	Annexin A4	GLGTDDNTLIR	16	9	0.0005	0.32	[83]
5	Q16853	AOC3	Membrane primary amine oxidase	YQLAVTQR	16	9	< 0.0001	0.29	
6	O95994	AGR2	Anterior gradientprotein 2 homolog	LPQTLSR	16	9	< 0.0001	0.29	[107]
7	O60218	AK1BA	Aldo–keto reductase family 1-member B10	SGDDLFPK	16	9	0.0152	0.27	[83]
8	Q13228	SBP1	Methanethiol oxidase	IYVVDVGSEPR	16	9	< 0.0001	0.26	[83]
9	P00167	CYB5	Cytochrome b5	FLEEHPGGEEVLR	16	9	< 0.0001	0.25	[83]
10	Q13228	SBP1	Methanethiol oxidase	LVLPSLISSR	14	5	0.0004	0.24	[83]
11	P51884	LUM	Lumican	ISNIPDEYFK	16	9	< 0.0001	0.18	
12	Q9UBX5	FBLN5	Fibulin-5	DQPFTILYR	16	9	< 0.0001	0.18	[52]
13	Q07507	DERM	Dermatopontin	YFESVLDR	16	9	< 0.0001	0.18	[55]
14	P51884	LUM	Lumican	ILGPLSYSK	16	9	< 0.0001	0.16	
15	P55083	MFAP4	Microfibril-associated glycoprotein 4	GFYYSLK	16	9	< 0.0001	0.16	[59]
16	P07585	PGS2	Decorin	VSPGAFTPLVK	16	9	< 0.0001	0.16	[49]
17	P51888	PRELP	Prolargin	NQLEEVPSALPR	16	9	< 0.0001	0.15	
18	Q07507	DERM	Dermatopontin	GATTTFSAVER	16	9	< 0.0001	0.14	[55]
19	P55083	MFAP4	Microfibril-associated glycoprotein 4	WTVFQK	14	5	< 0.0001	0.12	[59]
20	P00325	ADH1B	Alcohol dehydrogenase 1B	AAVLWEVK	14	5	< 0.0001	0.12	
21	P07585	PGS2	Decorin	ASYSGVSLFSNPVQYWEIQPSTFR	16	9	< 0.0001	0.11	[49]
22	P08294	SODE	Extracellular superoxide dismutase	VTGVVLFR	14	5	< 0.0001	0.11	
23	P23141	EST1	Liver carboxylesterase 1	FTPPQPAEPWSFVK	16	9	< 0.0001	0.08	[52, 83]
24	P20774	MIME	Mimecan	LTLFNAK	16	9	< 0.0001	0.07	[52]
25	P23141	EST1	Liver carboxylesterase 1	TVIGDHGDELFSVFGAPFLK	16	9	< 0.0001	0.06	[52, 83]
26	P20774	MIME	Mimecan	DFADIPNLR	16	9	< 0.0001	0.05	[52]

Proteins are ordered by descending FC. (A) upregulated proteins. (B) downregulated proteins

*Ca* Cancer, *CCA* cholangiocarcinoma, *Co* Control, *FC* Fold Change

### THBS2 expression in dCCA

**Fig. 6:**
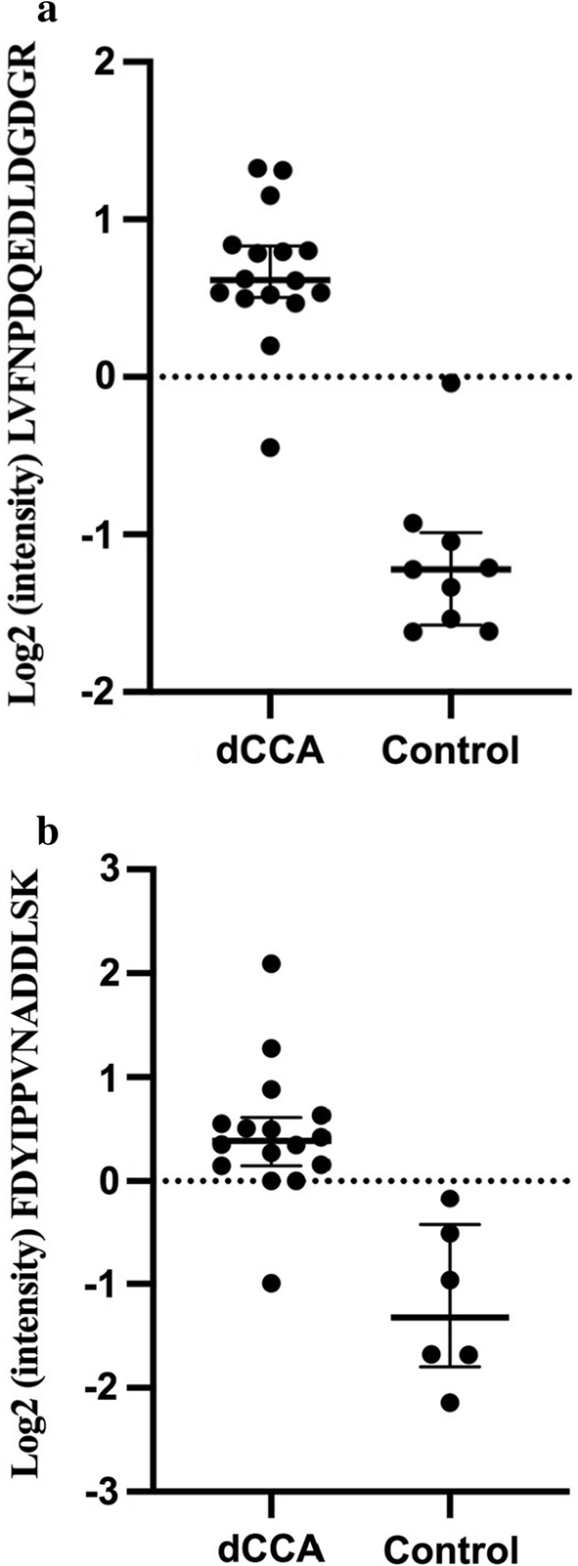
Boxplot of thrombospondin-2 intensities in distal cholangiocarcinoma and controls as quantified by PRM analysis. Peptides **a** FDYIPPVNADDLSK and **b** LVFNPDQEDLDGDGR

**Fig. 7:**
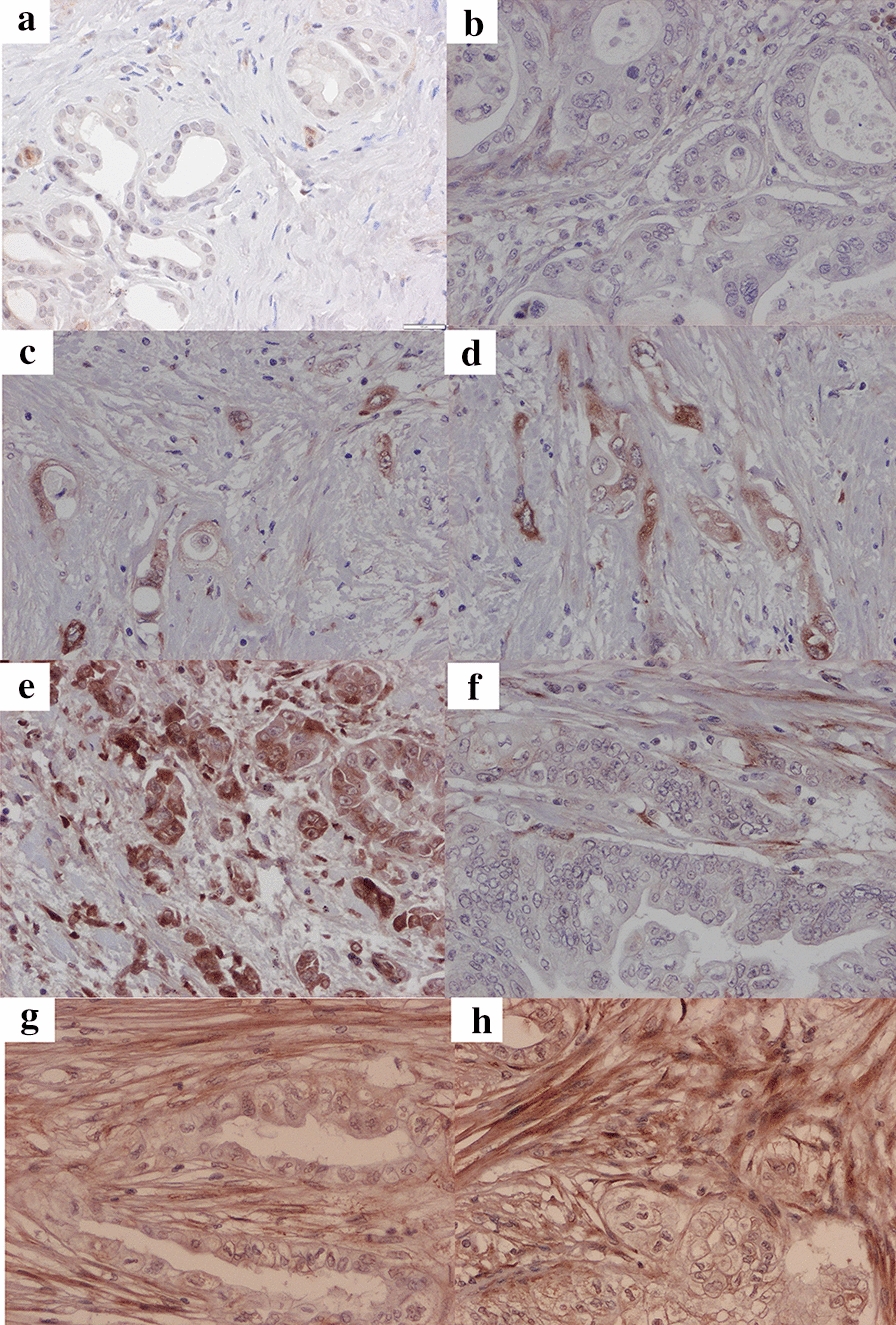
Representative immunohistochemical images at 200 × magnification showing THBS2 expression. Normal bile duct: **a** no expression. Distal cholangiocarcinoma: **b** no expression, **c** weak epithelial expression, **d** moderate epithelial expression, **e** strong epithelial expression, **f** weak stromal expression, **g** moderate stromal expression and **h** strong stromal expression

The most upregulated protein verified by PRM was THBS2 (Fig. 6), which had not previously been studied in CCA; THBS2 was selected for further validation using IHC. THBS2 showed a membranous/cytoplasmic staining pattern in epithelial cells and stromal fibroblasts. In the normal bile ducts epithelial THBS2 immunoreactivity was focally positive but predominantly negative. Weak focal stromal expression was also detectable but predominantly negative. Occasional intratumoral lymphocytic infiltration with positive THBS2 expression was noted but not evaluated further. Among the dCCA samples, epithelial immunoreactivity was absent in 6 samples (10%) and scored as 1+ in 30 samples (51%), 2+ in 13 samples (32%) and 3+ in 4 samples (7%). In dCCA stromal compartment, THBS2 expression was absent in 5 samples (8%) and scored as 1+ in 18 samples (31%), 2+ in 25 samples (42%) and 3+ in 11 samples (19%). A weak correlation between epithelial and stromal THBS2 immunoreactivity was observed (r_s_ = 0.32; P = 0.013). The proportion of samples positive for epithelial THBS2 decreased from 25 (96%) to 14 (54%) when primary tumors and paired lymph node metastasis samples were compared (P = 0.001). The proportion of samples positive for stromal THBS2 expression decreased from 25 (96%) to 18 (72%) when primary tumors and paired lymph node metastasis samples were compared (P = 0.031). Representative examples of IHC-staining are presented in Fig. 7.

**Fig. 8:**
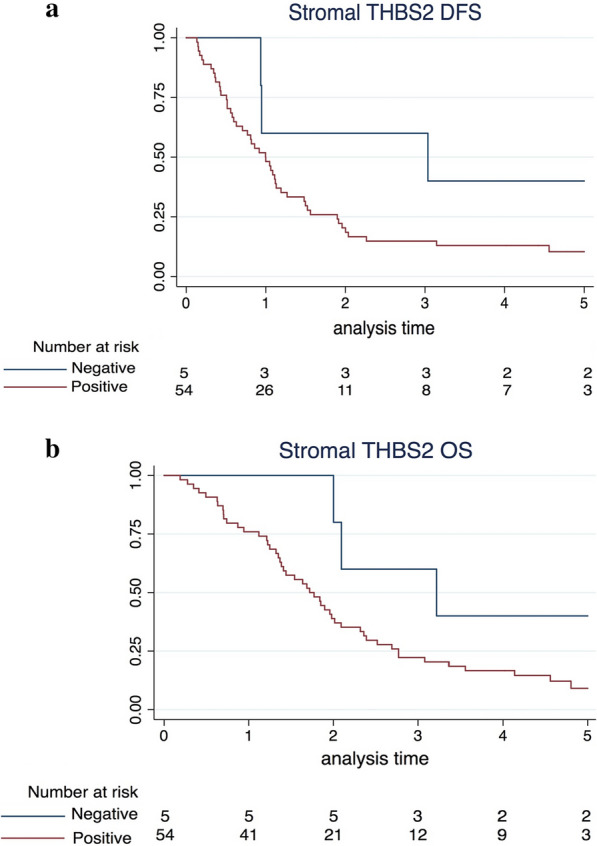
Kaplan–Meier curves of disease-free survival (**a**) and overall survival (**b**) stratified by stromal THBS2 expression

The distribution of clinicopathological variables between dCCA samples in the epithelial and stromal compartments with positive and negative THBS2 expression is presented in Additional file 8. Epithelial THBS2 positivity was associated with blood vessel invasion (P = 0.004), and stromal THBS2 positivity was associated with R1 resection (P = 0.011). There was no association between epithelial THBS2 and survival. Kaplan–Meier analysis revealed a trend towards lower DFS (P = 0.105) and OS (P = 0.079) in samples positive for THBS2 in the stromal compartment (Fig. 8). When confounding variables were adjusted with multivariable Cox proportional hazards regression (Additional file 9), positive stromal THBS2 expression was significantly associated with poor DFS (HR 3.95, 95% CI 1.09–14.3; P = 0.037) and tended to be associated with OS (HR 3.34, 95% CI 0.94–11.8; P = 0.062).

## Discussion

In this study, we identified DEPs between resected dCCA and normal bile duct samples using macrodissected archived FFPE tissues. A workflow using discovery LC–MS/MS followed by PRM verification of selected candidates was performed. In total, 46 proteins were successfully verified. Bioinformatic analysis highlighted alterations to the tumor reactive stroma (TRS) in dCCA. THBS2 was identified as upregulated in dCCA using MS and further validated to be upregulated in dCCA epithelium and stroma using IHC. There was a significantly lower proportion of epithelial and stromal THBS2 in paired lymph node metastases compared to primary tumors. However, stromal THBS2 expression in lymph node metastases was frequent (72%). Stromal THBS2 was significantly associated with poor DFS (HR 3.95, 95% CI 1.09–14.3; P = 0.037) when adjusted for confounding variables.

One of the characteristics of CCA tumor biology is the generation of a rich TRS [33]. The TRS consists of different cell types such as cancer-associated fibroblasts (CAFs) and tumor-associated macrophages; aberrant lymphatic vasculature; and a remodeled ECM, often with a dense desmoplastic reaction. Signaling interactions between cancer cells and the TRS play an important role in CCA carcinogenesis and treatment resistance [34]. GO analysis of DEPs from the discovery study revealed their enrichment in several TRS components. In addition, the majority of enriched pathways have been implicated in cancer-TRS interactions. This finding highlights the importance of the proteomic alterations to the TRS that occurs in dCCA.

Notably, using IHC, we found that the overexpression of THBS2 in dCCA identified by MS, could be due to both stromal and epithelial THBS2 overexpression. THBS2 is a primarily extracellular protein and has been shown to impact cellular functions such as angiogenesis [35], apoptosis [36], cytoskeletal organization and ECM remodeling [37]. THBS2 has been found to have both pro- and antitumorigenic functions and an association with both good and poor prognosis has been found in different cancers [38, 39]. In pancreatic cancer, THBS2 expression in cancer cells in vitro inhibited invasiveness through downregulation of matrix metalloproteinase-9 and urokinase-type plasminogen activator [40]. In contrast, stromal THBS2 expression in pancreatic stellate cells was found to promote cancer cell invasiveness in coculture experiments [41]. Discrepancies between the regulation and prognostic implications of epithelial and stromal THBS2 have been described [42, 43]. Recently, Kim et al. [44] demonstrated that plasma THBS2 is a promising diagnostic biomarker for pancreatic cancer. Given the biological similarities of dCCA and pancreatic cancer [45], further evaluation of THBS2 as a potential diagnostic biomarker of dCCA should be encouraged. We have not found any previous study in which the expression of THBS2 in paired lymph node metastases was investigated. Both epithelial THBS2 and stromal THBS2 were significantly less frequent in the lymph node metastases than in primary tumors. The loss of epithelial THBS2 during the dCCA metastatic process could be due to a tumor-suppressive function. Although significantly lower, stromal THBS2 expression was frequently retained in lymph node metastases (72%). The maintenance of THBS2 stromal cells through the metastatic process suggests a function of the THBS2 stroma in dCCA metastatic development. A large amount of stroma in lymph node metastases has previously been associated with poor survival in various cancers [46].

We note that among the most substantially downregulated proteins according to PRM verification were several proteins with a known TRS association. Proteins belonging to the small leucine-rich proteoglycan family such as decorin, lumican, prolargin, dermatopontin and mimecan were all substantially downregulated. Small leucine-rich proteoglycans are known to be involved in cellular proliferation, differentiation, survival, adhesion, migration, the inflammatory response and cancer development [47]. Decorin has a tumor-suppressive effect and has been extensively studied as a therapeutic target in epithelial cancers [48]. Furthermore, decorin was previously shown to be downregulated and associated with negative prognosis in eCCA. In addition, decorin treatment could reverse CCA proliferation, invasion and migration in vitro [49]. Mimecan is downregulated in several solid cancers and has a tumor-suppressive function through its interaction with epidermal growth factor receptor signaling and tumoral immune infiltration [50, 51]. Mimecan was also found to be downregulated in CCA [52]. Dermatopontin has been shown to be involved in transforming growth factor-β signaling, collagen fibrillogenesis, cell adhesion, and cell proliferation [53]. Additionally, dermatopontin has a tumor-suppressive effect [54] and was found to be downregulated in gallbladder cancer [55]. Prolargin has not been extensively studied in a cancer context however, low prolargin expression has been associated with poor survival in pancreatic cancer [56]. Lumican is known to modulate collagen fibrillogenesis and integrin signaling and has been shown to have a tumor-suppressive effect in several cancers [57]. To the best of our knowledge, neither prolargin nor lumican has been associated with dCCA. Other substantially downregulated proteins include microfibril-associated glycoprotein 4, fibulin-5 and extracellular superoxide dismutase. Microfibril-associated glycoprotein 4 is known to be downregulated in the majority of human cancers with prognostic significance and was predicted to impact cell proliferation and elastic fiber formation [58]. Microfibril-associated glycoprotein 4 was found to be downregulated in CAFs isolated from iCCA [59]. Fibulin-5 is downregulated in several human cancers and has a tumor-suppressive function mediated through its interaction with matrix metalloproteinases [60]. Furthermore, fibulin-5 was found to be downregulated in CCA [52]. Extracellular superoxide dismutase, an important enzyme in the antioxidant defense system, is frequently downregulated in cancer and has a tumor-suppressive role [61]; however, it has not been described in CCA biology. In addition, catalase another essential member of the antioxidant defense system was found to be downregulated [62].

When looking at the most upregulated proteins identified in our study, several proteins, such as protein S100-P [63, 64], carcinoembryonic antigen-related cell adhesion molecule 6 [65], keratin, type I cytoskeletal 17 [66, 67], thymidine phosphorylase [68, 69], heat shock protein 90 alpha/beta [70] and olfactomedin-4 [71, 72], are well established as upregulated proteins and tentative biomarkers in CCA. The correct identification and quantification of known biomarkers gives us confidence in the biological relevance of the proteins identified with our study design. Other substantially upregulated proteins without previous association with CCA include calponin-2, collagen triple helix repeat-containing protein 1 and ero1-like protein alpha. Calponin-2 is an actin cytoskeleton-associated regulatory protein that can inhibit cell proliferation and migration. Calponin-2 has not been extensively studied in cancer, however, it was found to be a prognostic factor and to have tumor-suppressive effects in pancreatic and prostate cancers [73, 74]. Collagen triple helix repeat-containing protein 1 is involved in the regulation of cell motility through the regulation of collagen deposition. It has an oncogenic effect and has been shown to be upregulated and associated with poor prognosis in several cancers [75]. Ero1-like protein alpha, which is important for disulfide bond formation in secreted molecules, is upregulated in several cancer forms and has been shown to play an important role in tumor-mediated immunosuppression [76].

Several previous studies have employed various MS-based proteomics approaches to identify biomarkers of cholangiocarcinoma through analysis of resected human tissues [72, 77–88]. The majority of these previous studies have investigated iCCA or a mixture of different CCA subtypes. A study by Maeda et al. [72] was the first study to analyze an eCCA-only cohort. To the best of our knowledge, our study represents the first proteomic characterization of a dCCA-only cohort.

Some limitations to the present study are noted. A small number of samples were analyzed. All material was retrospectively acquired and stored for various time periods prior to analysis. however, archival storage time of FFPE tissues has not been found to negatively impact MS based proteomics [89–91]. For the MS analysis, tumor and control samples were unmatched with regards to sex due to the limited number of tissues available for use as controls. Although we cannot exclude some bias as a result of this imbalance, the effect is likely minor. We choose to use normal tissue controls as compared to the more widely used and widely available morphologically normal tumor adjacent tissues since comparative studies have found molecular aberrations in normal tumor adjacent tissues compared to normal tissues [92]. This is hypothesized to be caused by field cancerization or microenvironment alterations influenced by the tumor [93]. Thus, normal controls can help identify additional biomarkers compared to normal tumor adjacent tissues [92]. In the PRM verification, a housekeeping normalization was used. Since there is currently no consensus on the most suitable housekeeping proteins in cholangiocarcinoma, we chose to use GAPDH and TUBB as housekeeping proteins since they are commonly used for this purpose and because suitable peptides were available in the discovery study data. However, expression of classic housekeeping genes was found to be less stable than assumed, especially in cancer tissues, which thus can be a source of bias [94]. IHC validation was also performed with a relatively small number of samples, particularly with regard to paired lymph node metastases. The survival analysis was underpowered for the detection of anything other than a large prognostic effect, especially as few cases were negative for THBS2. Notably, however, the effect size of the HR for stromal THBS2 was large.

## Conclusions

An MS-based workflow was used to identify and verify several proteins without a previous association with dCCA biology. The identified proteins can be further investigated to elucidate their function and potential as biomarkers in dCCA. THBS2 was validated as frequently expressed in the epithelium and stroma of dCCA. Stromal THBS2 is a potential prognostic marker; additionally, it was frequently retained in paired lymph node metastases. The use of stromal THBS2 as a prognostic marker in dCCA should be validated using separate larger cohorts, additionally the potential of THBS2 as a diagnostic biomarker in dCCA should be evaluated.

## Supplementary information

**Additional file 1.** Protein identification and quantification using MaxQuant and OpenMS.

**Additional file 2.** Clinicopathological data of analyzed samples.

**Additional file 3.** Total proteins identified by discovery LC-MS/MS.

**Additional file 4.** DEPs identified by discovery LC-MS/MS using PD, MaxQuant and OpenMS.

**Additional file 5.** Detailed enrichment analysis.

**Additional file 6.** Peptides included in the PRM spectral library.

**Additional file 7.** Peptides quantified by PRM.

**Additional file 8.** Distribution of clinicopathological variables in THBS2 positive and negative samples.

**Additional file 9.** Uni- and multivariable Cox proportional hazards regressions.

## Data Availability

The mass spectrometry proteomics data has been deposited to the ProteomeXchange Consortium via the PRIDE [95] partner repository with the dataset identifier PXD017906.

## Abbreviations

CCA: Cholangiocarcinoma
iCCA: Intrahepatic cholangiocarcinoma
eCCA: Extrahepatic cholangiocarcinoma
pCCA: Perihilar cholangiocarcinoma
dCCA: Distal cholangiocarcinoma
CA 19-9: Carbohydrate antigen 19-9
MS: Mass spectrometry
LC–MS/MS: Liquid phase separation on-line with tandem mass spectrometry
FFPE: Formalin-fixed paraffin-embedded
PRM: Parallel reaction monitoring
DEPs: Differentially expressed proteins
THBS2: Thrombospondin-2
IHC: Immunohistochemistry
AJCC: American Joint Committee on Cancer
R1-resection: Non-radical resection
DFS: Disease-free survival
OS: Overall survival
H&E: Hematoxylin and eosin
AMBIC: Ammonium bicarbonate
LC: Liquid chromatography
DDA: Data dependent acquisition
AGC: Automatic gain control
NCE: Normalized collision energy
PD: Proteome Discoverer
FDR: False discovery rate
GAPDH: Glyceraldehyde 3-phosphate dehydrogenase
TUBB: Tubulin beta chain
GO: Gene ontology
PCA: Principal component analysis
ECM: Extracellular matrix
CV: Coefficient of variation
TRS: Tumor reactive stroma
CAFs: Cancer-associated fibroblasts
