# Supplementary material for: Mass spectrometry-based analysis of formalin-fixed, paraffin-embedded distal cholangiocarcinoma identifies stromal thrombospondin-2 as a potential prognostic marker
Source: J Transl Med. 2020 Sep 4;18:343. doi: 10.1186/s12967-020-02498-3 (PMC7487897; doi:10.1186/s12967-020-02498-3)
Supplement: Supplementary file 1 — Additional file 1. Protein identification and quantification using MaxQuant and OpenMS. [file 12967_2020_2498_MOESM1_ESM.docx]

**MaxQuant**

MaxQuant version 1.5.6.5 (1) with integrated search engine Andromeda (2) was used. Protein identification was performed against the complete human proteome obtained from UniprotKB. As fixed modification carbamido-methylation of cystine was used, in addition N-terminal acetylation and oxidation of metionine residues was set as variable modifications. A maximum of 2 missed cleavages were allowed. A mass deviation of 6 ppm was allowed for the precursor ion and 20 ppm for fragment masses. The match between runs feature was applied with an alignment time windows of 30 s. The protein and peptide identification FDR were set to 0.01. 2 peptides per protein were required for identification, in additions proteins identified only by modified proteins were filtered out, and any protein matching to the reverse database or potential contaminant was likewise removed. The LFQ algorithm was used for quantification and normalization (3).

**OpenMS**

The converted mzml files (MSconvert, (4)) were analyzed by OpenMS v.2.0.0 (5) and TOPP (6) using X!tandem (7, 8) as search engine against the UniProt human database. The search included, cysteine carbamidomethylation as a fixed modification and methionine oxidation as a variable modification. The false discovery rate (FDR) was determined by searching a reverse database. The protein and peptide identification FDR were set to 0.01. Enzyme specificity was set to “trypsin” and one missed cleavage was allowed with a minimum of seven amino acids per identified peptide. Peptide identification was based on a search with an initial mass deviation of the precursor ion of up to 10 ppm, and the fragment mass deviation allowed was set to 20 ppm. A matching between runs feature was applied.

**References**

1. Cox J, Mann M. MaxQuant enables high peptide identification rates, individualized p.p.b.-range mass accuracies and proteome-wide protein quantification. Nature biotechnology. 2008;26(12):1367-72.

2. Cox J, Neuhauser N, Michalski A, Scheltema RA, Olsen JV, Mann M. Andromeda: a peptide search engine integrated into the MaxQuant environment. Journal of proteome research. 2011;10(4):1794-805.

3. Cox J, Hein MY, Luber CA, Paron I, Nagaraj N, Mann M. Accurate proteome-wide label-free quantification by delayed normalization and maximal peptide ratio extraction, termed MaxLFQ. Molecular & cellular proteomics : MCP. 2014;13(9):2513-26.

4. Kessner D, Chambers M, Burke R, Agus D, Mallick P. ProteoWizard: open source software for rapid proteomics tools development. Bioinformatics (Oxford, England). 2008;24(21):2534-6.

5. Sturm M, Bertsch A, Gropl C, Hildebrandt A, Hussong R, Lange E, et al. OpenMS - an open-source software framework for mass spectrometry. BMC bioinformatics. 2008;9:163.

6. Kohlbacher O, Reinert K, Gropl C, Lange E, Pfeifer N, Schulz-Trieglaff O, et al. TOPP--the OpenMS proteomics pipeline. Bioinformatics (Oxford, England). 2007;23(2):e191-7.

7. Craig R, Beavis RC. TANDEM: matching proteins with tandem mass spectra. Bioinformatics (Oxford, England). 2004;20(9):1466-7.

8. Craig R, Beavis RC. A method for reducing the time required to match protein sequences with tandem mass spectra. Rapid communications in mass spectrometry : RCM. 2003;17(20):2310-6.
